# Determining What Changed Japanese Suicide Mortality in 2020 Using Governmental Database

**DOI:** 10.3390/jcm10215199

**Published:** 2021-11-07

**Authors:** Ryusuke Matsumoto, Eishi Motomura, Kouji Fukuyama, Takashi Shiroyama, Motohiro Okada

**Affiliations:** Department of Neuropsychiatry, Division of Neuroscience, Graduate School of Medicine, Mie University, Tsu 514-8507, Japan; matsumoto-r@clin.medic.mie-u.ac.jp (R.M.); motomura@clin.medic.mie-u.ac.jp (E.M.); k-fukuyama@clin.medic.mie-u.ac.jp (K.F.); takashi@clin.medic.mie-u.ac.jp (T.S.)

**Keywords:** suicide mortality, Japan, COVID-19, gender, region, age, motive, means, household

## Abstract

The pandemic of 2019 novel coronavirus disease (COVID-19) caused both COVID-19-related health hazards and the deterioration of socioeconomic and sociopsychological status due to governmental restrictions. There were concerns that suicide mortality would increase during the COVID-19 pandemic; however, a recent study reported that suicide mortality did not increase in 21 countries during the early pandemic period. In Japan, suicide mortality was reduced from 2009 to 2019, but both the annual number of suicide victims and the national suicide mortality rates in 2020 increased compared to that in 2019. To clarify the discrepancy of suicide mortality between the first and second half of 2020 in Japan, the present study determines annual and monthly suicide mortality disaggregated by prefectures, gender, age, means, motive, and household factors during the COVID-19 pandemic and pre-pandemic periods using a linear mixed-effects model. Furthermore, the relationship between suicide mortality and COVID-19 data (the infection rate, mortality, and duration of the pandemic) was analysed using hierarchal linear regression with a robust standard error. The average of monthly suicide mortality of both males and females in all 47 prefectures decreased during the first stay-home order (April–May) (females: from 10.1–10.2 to 7.8–7.9; males: from 24.0–24.9 to 21.6 per 100,000 people), but increased after the end of the first stay-home order (July–December) (females: from 7.5–9.5 to 10.3–14.5; males: from 19.9–23.0 to 21.1–26.7 per 100,000 people). Increasing COVID-19-infected patients and victims indicated a tendency of suppression, but the prolongation of the pandemic indicated a tendency of increasing female suicide mortality without affecting that of males. Contrary to the national pattern, in metropolitan regions, decreasing suicide mortality during the first stay-home order was not observed. Decreasing suicide mortality during the first stay-home order was not observed in populations younger than 30 years old, whereas increasing suicide mortality of populations younger than 30 years old after the end of the first stay-home order was predominant. A decrease in suicide mortality of one-person household residents during the first stay-home order was not observed. The hanging suicide mortality of males and females was decreased and increased during and after the end of the first stay-home orders, respectively; however, there was no decrease in metropolitan regions. These results suggest that the suicide mortality in 2020 of females, younger populations, urban residents, and one-person household residents increased compared to those of males, the elderly, rural residents, and multiple-person household residents. Therefore, the unexpected drastic fluctuations of suicide mortality during the COVID-19 pandemic in Japan were probably composed of complicated reasons among various identified factors in this study, and other unknown factors.

## 1. Introduction

The 2019 novel coronavirus disease (COVID-19) had globally infected more than 242 million people and contributed to over 4.9 million deaths as of 25 October 2021 [1]. In Japan, more than 1.7 million people were infected with COVID-19, and more than 18,000 lives were lost due to COVID-19 as of 25 October 2021 [1,2]. Even today, when vaccines are more widespread, the COVID-19 pandemic continues. In addition to COVID-19-related health hazards, governmental COVID-19 restrictions have impacted lives and lifestyles, resulting in the deterioration of socioeconomic and sociopsychological status. A number of studies expressed sociopsychological concerns that the COVID-19 pandemic has encouraged isolation, fear, marginalisation, psychiatric disorders, domestic abuse, and intimate partner violence [3,4,5,6,7]. Social conditions that force the drastic modification of lifestyles and the economy play important roles in increasing suicide mortality [8,9]. Contrary to our expectations, a recent study revealed that the risk of suicide of 21 countries could not be detected during the early COVID-19 pandemic periods, including Japan [10]. However, an extended observation period reported that the prolongation of the COVID-19 pandemic periods increased suicide mortality in some countries and areas, such as Japan, Puerto Rico, and Vienna (Austria) [10].

**Figure 1:**
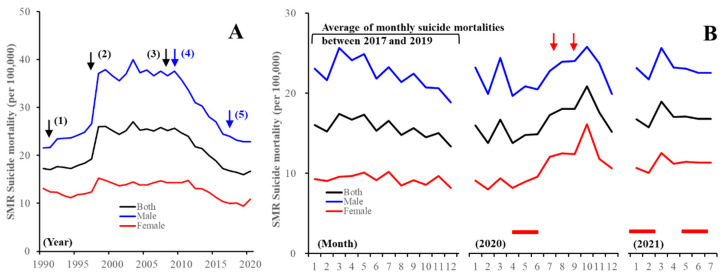
(**A**) Annual and (**B**) monthly suicide mortality in Japan. Ordinates indicate standardised mortality ratio (SMR) of suicide mortality of males and females (black lines), males (blue line), and females (red line) in Japan per 100,000 people. (**A**) Black arrows (1–3) indicate the collapse of the asset bubble, the Asian economic crisis, and the 2008 global financial crisis, respectively. Blue arrows (4) and (5) indicate the contribution of the Emergency Fund to Enhance Community-Based Suicide Countermeasures and the introduction of the Revised Basic Act on Suicide Prevention, respectively. (**B**) (left) Average SMR suicide mortalities during 2017–2019. (middle, right) Monthly SMR suicide mortality in 2020 and 2021, respectively. Red columns, period of 2019 novel coronavirus disease (COVID-19) stay-home orders; red arrows, mass media reports regarding celebrity suicides.

Several analytical studies that have utilised governmental suicide databases covering the entire population reported that the impact of the COVID-19 pandemic on suicide mortality in Japan may change over time and could have various different targets [11,12]. In Japan, suicide mortality was steadily decreasing between 2009 and 2019, but increasing in 2020: from 20,169 (males: 14,078, females: 6091) to 20,919 (males: 13,943, females: 6976) (Figure 1) [13]. Analysis of gender-related dynamics of suicide mortality indicated confusing results [14,15,16,17,18,19]. The annual suicide mortality of males decreased compared to that in 2019, but that of females increased [15,16,17,18,19] (Figure 1). In various Asian countries including Japan, where an adverse effect of the economic crisis on suicide mortality was detected around the 2008 Asian economic crisis, the impact of the economic crisis on the suicide mortality of males and elderly populations was greater compared to that of females and younger populations (Figure 1A) [8,20]. According to this evidence, comprehensive suicide prevention programmes in Japan reduced suicide mortality due to targeting males and elderly populations [21,22,23,24,25]; however, the economic recession and increasing suicide mortality in Japan were not temporally related between 2009 and 2019 (Figure 1A). Therefore, increasing suicide mortality in 2020 was uninterpretable using previous findings. The Ministry of Health, Labour, and Welfare (MHLW) speculated that the increasing female suicide mortality was probably induced by mass media reports associated with the suicide and death of celebrities with COVID-19, and increasing domestic violence, as evidenced by the WHO guideline [26]. MHLW announced nine alerts in the mass media [27,28] from September 2020 to September 2021, issuing a warning that reporting should be conducted according to suicide reporting guidelines “Preventing suicide: a resource for media professionals” [26].

The first modern global pandemic was the influenza pandemic (Spanish flu) between 1918 and 1920. As well as the Spanish flu [29,30], other infectious pandemics whose impacts on suicide mortality have been analysed include severe acute respiratory syndrome (SARS) [31,32,33] and COVID-19. Regarding the Spanish flu pandemic, suicide mortality increased after the first pandemic phase in 1919, but an increase was not observed after the second pandemic phase [29]; however, details of the impacts of the Spanish flu pandemic are not clear due to suppressed reporting of the pandemic during World War I [30]. Regarding the SARS pandemic in Hong Kong, a persistently increasing suicide mortality rate (over 2 years) compared to the pre-pandemic period was detected [31,32,33]. Increasing and decreasing (valley of suicide) suicide mortality rates were detected that were synchronised with the peak of the SARS pandemic, as well as 2 months after the peak, but these fluctuations were limited in older females [31,32]. Notably, feeling disconnected was a more common problem in individuals who were identified as having committed suicide in relation to SARS than in those who were identified as having committed suicide for other reasons [33,34]. However, there is little information and evidence regarding the time-dependent changes in suicide mortality induced by the COVID-19 pandemic in Japan. Thus, a number of reports speculated on the basis of evidence relating to previous public health emergencies arising from natural disasters [7,34,35,36,37,38]. Short-term decreasing suicide rates in the immediate aftermath of natural disasters are called the “honeymoon period” or “pulling together” phenomenon [37,38]. Regional panel data analysis in Japan revealed that, when damage caused by natural disasters is extremely severe, suicide mortality tends to increase in the immediate aftermath of the disaster and several years later; however, when the damage by natural disasters is less severe, suicide mortality rates tend to decrease after the disasters, especially one or two years later [36]. Taken together with previous findings, the time-dependent kinetics of Japanese suicide mortality in 2020 is more similar to the tendency induced by less severe natural disasters rather than that by severe natural disasters or an economic crisis. The COVID-19 pandemic has imposed a slow and long-term burden on society and individuals compared to severe natural disasters such as earthquakes and tsunamis. Indeed, increasing suicide mortality rates are concerning due to the prolongation of the COVID-19 pandemic, since the rate of suicidal ideations during the COVID-19 pandemic is higher than that reported in studies on the general population during the pre-pandemic period [39]. Therefore, the long-lasting burden on societies and individuals induced by the COVID-19 pandemic is fundamentally different from that of previous natural disasters and economic crises. John et al. reported that “any change in the risk of suicide associated with COVID-19 is likely to be dynamic” [40]. Therefore, to clarify who, when, how, and why individuals died by suicide in Japan during the COVID-19 pandemic, this study determines the change in suicide mortality during the pandemic compared to the pre-pandemic period using a governmental database.

## 2. Materials and Methods

### 2.1. Dependent and Independent Variables

The numbers of suicide victims of 47 prefectures in Japan from January 2017 to June 2021 were obtained from Basic Data on Suicide in the Region (BDSR) in a national database of the MHLW [13]. BDSR published the numbers of suicide victims disaggregated by prefecture (47 prefectures), gender, suicide motives (health, family, economy, romance, employment, and school-related motives), suicide means (hanging, charcoal burning, jumping, poisoning, and throwing), household (multiple-person and one-person), and ages (0–19 (10s), 20–29 (20s), 30–39 (30s), 40–49 (40s), 50–59 (50s), 60–69 (60s), 70–79 (70s), and over 80 years old (80s)) [13]. The numbers of suicide victims in BDSR between January 2017 and December 2020 were definitive values, but as of September 2021, suicide victims between January and June 2021 are provisional values (final definitive value will be published in March 2022). Prefectural population data were obtained from the Regional Statistics Database (RSD) of the System of Social and Demographic Statistics of the Statistics Bureau of the Ministry of Internal Affairs and Communications (SBMIAC) [41].

Annual standardised mortality of suicide per 100,000 people (SMR-S) was calculated by dividing the numbers of suicide victims per prefecture by the prefectural population (denominator) of the same years. Monthly SMR-S, which was also calculated by dividing monthly numbers of suicide victims per prefecture by the prefectural population of the same years, was converted annually and adopted for statistical analysis. Annual and monthly age, gender, and prefecture disaggregated SMR-S were also derived from age, gender, and prefecture disaggregated numbers of suicide victims (numerator) in BDSR [13], and age, gender, and prefecture disaggregated population exposure (denominator) [41] in RDS [22]. BDSR data were classified by the number of suicides into six types of suicide motives (health-, family-, economy-, romance-, employment-, and school-related problems), five types of suicidal means (hanging, poisoning, charcoal burning, jumping, and throwing), and household conditions (one- and multiple-person households) [13]. Suicide victims in each region were counted by the jurisdiction of local police stations. The police investigate personal characteristics and background factors of each suicide victim. The results of this investigation contain a number of motives for suicide, and these motives were compared to previously compiled suicide motive lists. Lastly, the investigation identifies the possible motive for suicide on the basis of evidence, suicide notes, or other documentation such as medical certificates, clinical recording, and the testimony of the surviving family [42,43].

The monthly numbers of infected individuals with COVID-19 and death caused by COVID-19 were obtained from the Database of the National Institute of Infectious Diseases [2] and Sapporo Medical University School of Medicine [44]. The monthly COVID-19 infection ratio per 100,000 people (SCR) and the mortality ratio caused by COVID-19 per 100,000 people (SMR-C) were calculated by dividing the numbers of infected individuals and deaths caused by COVID-19 per prefecture by the prefectural population (denominator) of the same years. The monthly duration of the COVID-19 pandemic (DCP) was set as the monthly basis, with March 2020 as one month, since the SCR was drastically increased in March 2020 (first Japanese patients with COVID-19 and victims who had never travelled to China were confirmed in January and February 2020, respectively).

### 2.2. Statistical Analysis

The annual SMR-S between the pre-pandemic (between 2017 and 2019) and COVID-19 pandemic (between 2020 and 2021) periods was compared using a linear mixed-effects model using BellCurve for Excel v.3.2 (Social Survey Research Information Co., Ltd., Tokyo, Japan) [45,46,47]. Monthly SMR-S between the COVID-19 pandemic period (between January 2020 and June 2021) and the average of SMR-S at the same month pre-pandemic (between January 2017 and December 2019) were also compared by linear mixed-effects model using BellCurve for Excel v.3.2. When the F value of the linear mixed-effects model was significant (*p* < 0.05), data were analysed by Tukey’s multiple comparison test. The governmental guideline for suicide prevention in Japan was stipulated in the General Policies for Comprehensive Measures against Suicide, which was revised in 2017. This revised guideline required prefectures to majorly improve scientific evidence-based regional suicide prevention programmes. Therefore, the pre-pandemic period was set between 2017 and 2019. The present study analysed the impact of SCR (monthly standardised COVID-19 infection ratio), SMR-C (monthly standardised mortality ratio caused by COVID-19), and DCP (duration of COVID-19 pandemic) on monthly SMR-S (between March 2020 and June 2021) using a hierarchical linear regression model with robust standard error (HLM7, Scientific Software International, Skokie, IL, USA) [23,24].

First, both linear mixed-effects and hierarchical linear regression models were analysed in all 47 prefectures. The target regions of the first governmental stay-home order (between April and May 2020) were major metropolitan regions in Japan, such as Tokyo, Saitama, Chiba, and Kanagawa, the Kansai area (Osaka, Kyoto, and Hyogo, Japan), the Chukyo area (Aichi), the Fukuoka area (Fukuoka), and the Sapporo area (Hokkaido, Japan). Second, both linear mixed-effects and hierarchical linear regression models were analysed in metropolitan regions as serious COVID-19 infection areas.

## 3. Results

### 3.1. Suicide Mortality Disaggregated by Gender

**Figure 2:**
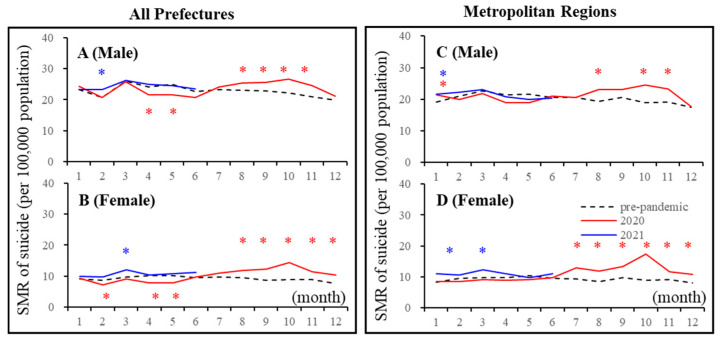
Temporal fluctuations of monthly standardised suicide mortalities (SMR-S) during COVID-19 pandemic period (2020–2021) compared to the average of the same month of SMR-S during the pre-pandemic period (2017–2019). Dotted black, red, and blue lines indicate the average of SMR-S for (**A**) males, (**B**) females of all 47 prefectures, and (**C**) males and (**D**) females of metropolitan regions. Ordinates indicate the SMR-S (per 100,000 people), and abscissas indicate the month. * *p* < 0.05, significant change using a linear mixed-effects model with Tukey’s multiple comparison. Red and blue asterisks indicate significant changes in SMR-S in 2020 and 2021, respectively, compared to the average SMR-S of the same month during the pre-pandemic period.

**Figure 3:**
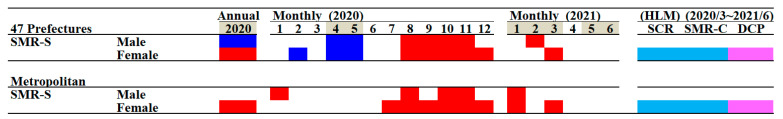
Comparison of annual and monthly standardised suicide mortalities (SMR-S) during COVID-19 pandemic period compared to the average of the same month of SMR-S during the pre-pandemic period (2017–2019), and the impact of the COVID-19 pandemic on SMR-S. Blue and red columns indicate significant decreasing and increasing suicide mortalities using a linear mixed-effects model with Tukey’s multiple comparison (*p* < 0.05). Light blue and red columns indicate significant decrease and increase factors against SMR-S using hierarchical linear model with robust standard error (*p* < 0.05). SMR-S: standardised suicide mortality per 100,000 people. SCR: standardised infection with COVID-19 per 100,000 people. SMR-C: standardised mortality caused by COVID-19 per 100,000 people. DCP: during COVID-19 pandemic.

Linear mixed-effect models detected the significantly different annual and monthly SMR-S of all 47 prefectures between COVID-19 pre-pandemic (between 2017 and 2019) and pandemic (between 2020 and 2021) periods. In all 47 prefectures, the annual SMR-S of females was increased in 2020, but that of males was decreased compared to average SMR-S during the pre-pandemic period (Figure 2 and Figure 3). In 2020, the monthly SMR-S of males and females decreased during the first stay-home order (between April and May 2020) compared to the average SMR-S at the same month during the pre-pandemic period (Table 1, Figure 2 and Figure 3). The monthly SMR-S of both males and females increased between the first and second stay-home orders (between August and December 2020) compared to the average SMR-S in the same month during the pre-pandemic period (Table 1, Figure 2 and Figure 3). In 2021, the trends of the monthly SMR-S of males and females were different from those in 2020. The SMR-S of both males and females during the second stay-home order (between January and March 2021) increased, but there were no differences in that of both males and females during the third stay-home order (between May and July 2021) compared to the pre-pandemic period (Table 1, Figure 2 and Figure 3).

These results indicate that significant fluctuations were observed in the suicide mortality of both males and females in 2020, which were classified into two phases: decreasing suicide during the first stay-home order and increasing suicide between the first and second stay-home orders. The suicide mortality of males and females in 2021 is expected to be slightly higher than that in pre-pandemic periods, but it seems to be stabilising, at least compared to the fluctuations of suicide mortality in 2020. The response of suicide mortality to the stay-home order was probably attenuated in a frequency-dependent manner of announcements of the stay-at-home orders.

In five major metropolitan regions that were the most severe pandemic regions (capital, Kansai, Chukyo, Fukuoka, and Sapporo metropolitan regions), the linear mixed-effect model detected significantly different annual and monthly SMR-S between the pre-pandemic (between 2017 and 2019) and pandemic periods (between 2020 and 2021) (Table 1, Figure 2 and Figure 3). The annual SMR-S of females in 2020 increased, but that of males did not change compared to the average SMR-S of five metropolitan regions during the pre-pandemic period (Table 1, Figure 2 and Figure 3). In 2020, the monthly SMR-S of males and females did not change during the first stay-home order compared to the average SMR-S during the pre-pandemic period; however, similar to all 47 prefectures, the monthly SMR-S of males and females increased between the first and second stay-home orders compared to the average SMR-S during the pre-pandemic period (Table 1, Figure 2 and Figure 3). In 2021, the trends of the monthly SMR-S of males and females in 2021 were different from those in 2020, but similar to the trends of all 47 prefectures (Table 1, Figure 2 and Figure 3). The monthly SMR-S of males and females during the second stay-home order increased, whereas the SMR-S of both males and females during the third stay-home order was almost equal to that in the pre-pandemic period (Table 1, Figure 2 and Figure 3).

Therefore, fluctuations in suicide mortality in metropolitan regions were quite different in all 47 prefectures, since no decrease in the suicide mortality of males and females in metropolitan regions during the first stay-home order was observed. Over the past decade, monthly suicide mortality in Japan has been high in the first quarter and decreasing in the following periods [13,18,48]; however, the fluctuation pattern of suicide mortality in 2020 was a variation against the traditional pattern, as it increased in the third and fourth quarters (Figure 1 and Figure 2).

Hierarchical linear model analysis detected a significant impact of SCR, SMR-C, and DCP on SMR-S during the pandemic period (between March 2020 and June 2021). The SMR-S of females in both all 47 prefectures and metropolitan regions was negatively related to SCR and SMR-C, but positively related to DCP; however, the SMR-S of males in both all 47 prefectures and metropolitan regions was not related to SCR, SMR-C, or DCP. These results suggest that the increase in patients with COVID-19 (SCR) and victims of COVID-19 (SMR-C) contributed to a reduction in the suicide mortality of females, but the prolongation of the pandemic led to increased female suicide mortality. The suicide mortality of males, on the other hand, is probably less sensitive to the influence of any data associated with COVID-19.

### 3.2. Suicide Mortality Disaggregated by Age

To identify the major factors of decreasing SMR-S during the first stay-home order and increasing SMR-S between the first and second stay-home orders, SMR-S disaggregated by age and gender factors was analysed using linear mixed-effects and hierarchical linear models.

**Figure 4:**
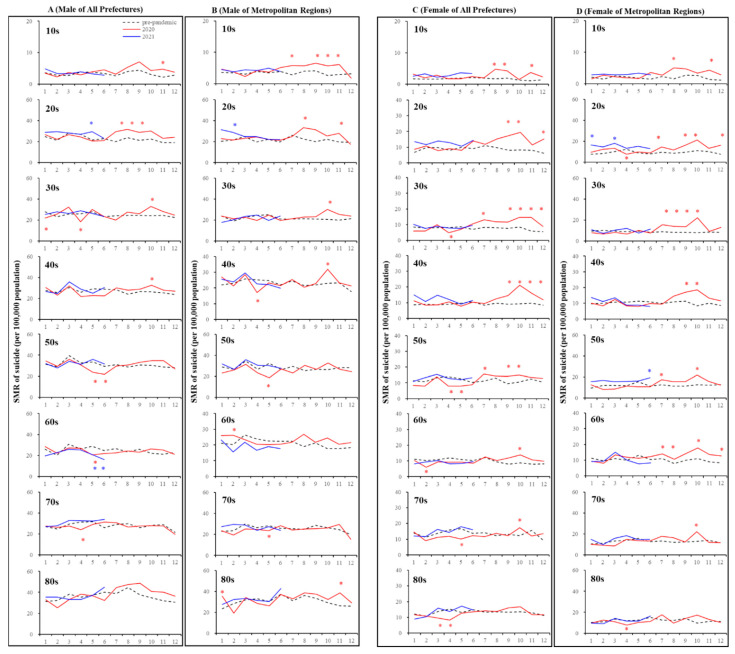
Temporal fluctuations in monthly SMR-S disaggregated by gender, age, and regional factors during the COVID-19 pandemic period (2020–2021) compared to the average of SMR-S during the same month during the pre-pandemic period (2017–2019). Dotted black, red, and blue lines indicate the average of SMR-S for (**A**) males from all 47 prefectures, (**B**) males from metropolitan regions, (**C**) females from all 47 prefectures, and (**D**) females from metropolitan regions. Ordinates indicate the SMR-S (per 100,000 people), and abscissas indicate the month. * *p* < 0.05, significant change using a linear mixed-effects model with Tukey’s multiple comparison. Red and blue asterisks indicate significant changes in SMR-S in 2020 and 2021, respectively, compared to the average SMR-S of the same month during the pre-pandemic period.

**Figure 5:**
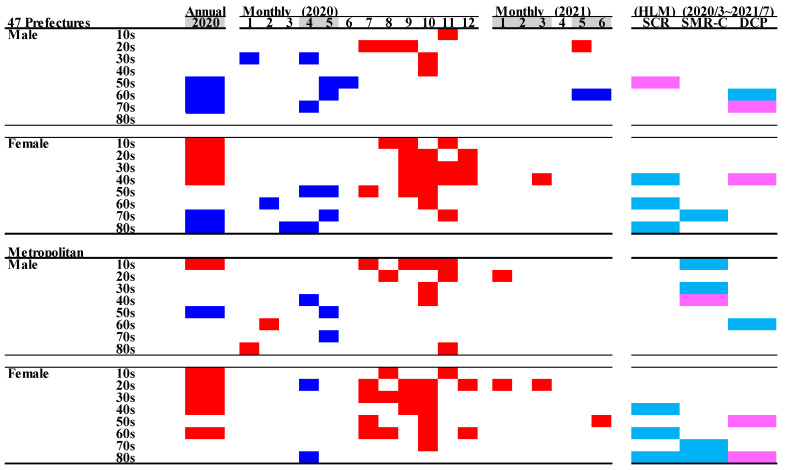
Comparison of annual and monthly SMR-S disaggregated by age during the pandemic period compared to the average SMR-S of the same month during the pre-pandemic period, and impact of COVID-19 pandemic on SMR-S in Japan. Blue and red columns indicate significant decreasing and increasing suicide mortality using a linear mixed-effects model with Tukey’s multiple comparison (*p* < 0.05). Light blue and red columns indicate significant decreasing and increasing factors against SMR-S using hierarchical linear model with robust standard error (*p* < 0.05). SMR-S: standardised suicide mortality per 100,000 people. SCR: standardised infection with COVID-19 per 100,000 people. SMR-C: standardised mortality caused by COVID-19 per 100,000 people. DCP: during COVID-19 pandemic.

In all 47 prefectures, in spite of decreasing monthly SMR-S in males during the first stay-home order, increasing monthly SMR-S in males over 50 between the first and second stay-home orders was not detected. The annual SMR-S of elderly (70s and 80s) females in 2020 decreased, whereas the annual SMR-S of females younger than 40 increased compared to the average annual SMR-S of the pre-pandemic period (Figure 4 and Figure 5). In metropolitan regions, decreasing and increasing annual SMR-S in 2020 was not observed except for the SMR-S of males in their 50s and 10s, respectively (Figure 4 and Figure 5).

In all 47 prefectures, the kinetics of the monthly SMR-S of males and females decreased during the first stay-home order, but the monthly SMR-S of males and females was increased between the first and second stay-home orders compared to the average SMR-S for the same month during the pre-pandemic period (Figure 4 and Figure 5). The tendency of decreasing SMR-S during the first stay-home order was more predominant in older populations in both males (30s–70s) and females (50s–80s) compared to younger populations. Increasing SMR-S between the first and second stay-home orders was detected in younger populations in males (10s–40s), but not in older males (50s–80s) (Figure 4 and Figure 5). In contrast, a decrease during the first stay-home order and an increase between the first and second stay-home orders in the SMR-S of females were detected in a wide range of ages of females (30s–70s), but a decrease during the first stay-home order and an increase between the first and second stay-home orders in SMR-S were not observed in younger (10s and 20s) and elderly (80s) females, respectively (Figure 4 and Figure 5).

In metropolitan regions, the age-dependent fluctuations of males’ SMR-S were more pronounced, since the fluctuation of younger males (10s–30s) increased between the first and second stay-home orders without a decrease during the first stay-home order. Contrary to males, an increased SMR-S of wide-range-age females was observed, whereas a decrease in the SMR-S of females during the first stay-home order was not detected except for females in their 20s and 80s (Figure 4 and Figure 5).

In SMR-S disaggregated by only the gender factor, the polarised responses of SMR-S to COVID-19-related data, SCR, SMR-C, and DCP were slightly weakened, using SMR-S disaggregated by gender with age factors by the hierarchical linear model (Figure 5). A positive relationship between SMR-S and DCP was still detected in females over 30 in all 47 prefectures, whereas a negative impact of SCR and SMR-C on SMR-S was not detected in females except for those in their 70s (Figure 5). Furthermore, in metropolitan regions, a positive impact of DCP on SMR-S was detected in only females in their 40s and 80s, whereas a negative impact of SCR and SMR-C on SMR-S was detected in females in their 60s–80s. In all 47 prefectures and metropolitan regions, a significant responsiveness of males’ SMR-S to COVID-19-related data, SCR, SMR-C, and DCP was also detected, but with less consistent results (Figure 5). Nevertheless, the impact of COVID-19-related data, SCR, SMR-C, and DCP on the SMR-S of males was negligible compared to that of females.

### 3.3. Suicide Mortality Disaggregated by Household Condition

**Figure 6:**
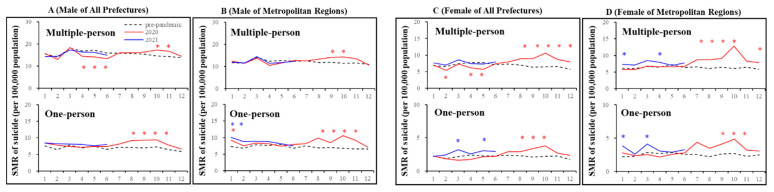
Temporal fluctuations in monthly SMR-S disaggregated by gender, household condition, and regional factors during the COVID-19 pandemic period (2020–2021) compared to the average of SMR-S for the same month during the pre-pandemic period (2017–2019). Dotted black, red, and blue lines indicate the average of SMR-S for (**A**) males from all 47 prefectures, (**B**) males from metropolitan regions, (**C**) females from all 47 prefectures and (**D**) females from metropolitan regions. Ordinates indicate the SMR-S (per 100,000 people), and abscissas indicate the month. * *p* < 0.05, significant change using a linear mixed-effects model with Tukey’s multiple comparison. Red and blue asterisks indicate significant changes in SMR-S in 2020 and 2021, respectively, compared to the average SMR-S of the same month during the pre-pandemic period.

**Figure 7:**
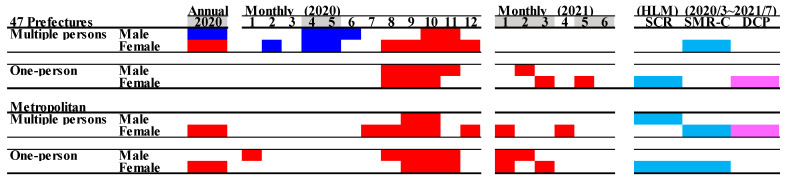
Comparison of annual and monthly SMR-S disaggregated by household condition during the pandemic period to average SMR-S of the same month during the pre-pandemic period, and impact of COVID-19 pandemic on SMR-S in Japan. Blue and red columns indicate significant decreasing and increasing suicide mortality using a linear mixed-effects model with Tukey’s multiple comparison (*p* < 0.05). Light blue and red columns indicate significant decreasing and increasing factors against SMR-S using hierarchical linear model with robust standard error (*p* < 0.05). SMR-S: standardised suicide mortality per 100,000 people. SCR: standardised infection with COVID-19 per 100,000 people. SMR-C: standardised mortality caused by COVID-19 per 100,000 people. DCP: during COVID-19 pandemic.

In all 47 prefectures, the annual SMR-S in 2020 for multiple-person household resident males and females decreased and increased, respectively; however, the SMR-S for one-person household resident males nor females did not change (Figure 6 and Figure 7). The monthly SMR-S of multiple-person household resident males and females decreased during the first stay-home order (Figure 6 and Figure 7). Contrary to multiple-person residents, the monthly SMR-S of one-person household residents (both males and females) did not change during the first stay-home order; however, between the first and second stay-home orders, the monthly SMR-S of multiple- and one-person resident males and females increased (Figure 6 and Figure 7). The monthly SMR-S of one-person household resident males and females increased during the second or third stay-home order, whereas an increase was not detected in multiple-person household resident males or females during 2021 (Figure 6 and Figure 7).

In metropolitan regions, a decrease in the monthly SMR-S of multiple- and one-person households resident males and females was not observed during the first stay-home order (Figure 5). Between the first and second stay-home orders, the monthly SMR-S of multiple- and one-person household resident males and females increased. Similar to all 47 prefectures, the monthly SMR-S of one-person household resident males and females increased during the second stay-home order. The monthly SMR-S of multiple-person household resident females increased in 2021, but that of males did not in 2021 (Figure 5).

The hierarchical linear model detected a significantly positive impact of DCP on the SMR-S of all females. There was a negative impact of SMR-C on the SMR-S of multiple-person household resident females in both all 47 prefectures and metropolitan regions, and one-person household resident females in metropolitan regions; however, SCR was negatively related to only the SMR-S of one-person household resident females in all 47 prefectures. The SMR-S of multiple-person household resident males in metropolitan regions was negatively related to SCR, whereas a relationship of other SMR-S of males with COVID-19-related data was not detected (Figure 5).

### 3.4. Suicide Mortality Disaggregated by Suicide Means

**Figure 8:**
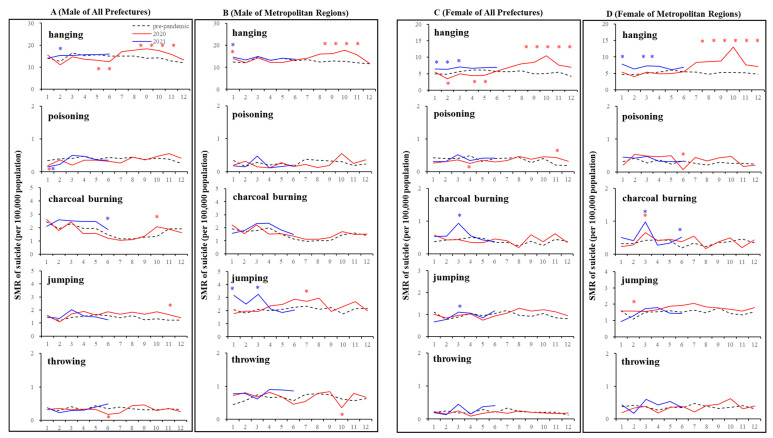
Temporal fluctuations in monthly SMR-S disaggregated by gender, suicidal means, and regional factors during COVID-19 pandemic period (2020–2021) compared to the average of SMR-S in the same month during the pre-pandemic period (2017–2019). Dotted black, red, and blue lines indicate the average of SMR-S for (**A**) males from all 47 prefectures, (**B**) males from metropolitan regions, (**C**) females from all 47 prefectures, and (**D**) females from metropolitan regions. Ordinates indicate the SMR-S (per 100,000 people), and abscissas indicate the month. * *p* < 0.05, significant change using a linear mixed-effects model with Tukey’s multiple comparison. Red and blue asterisks indicate significant changes in SMR-S in 2020 and 2021, respectively, compared to the average SMR-S of the same month during the pre-pandemic period.

**Figure 9:**
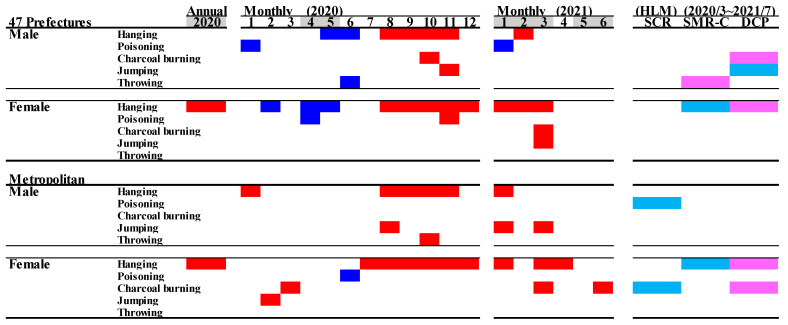
Comparison of annual and monthly SMR-S disaggregated by suicide means during the pandemic period to average SMR-S of the same month during pre-pandemic period, and impact of COVID-19 pandemic on SMR-S in Japan. Blue and red columns indicate significant decreasing and increasing suicide mortality using a linear mixed-effects model with Tukey’s multiple comparison (*p* < 0.05). Light blue and red columns indicate significant decreasing and increasing factors against SMR-S using hierarchical linear model with robust standard error (*p* < 0.05). SMR-S: standardised suicide mortality per 100,000 people. SCR: standardised infection with COVID-19 per 100,000 people. SMR-C: standardised mortality caused by COVID-19 per 100,000 people. DCP: during COVID-19 pandemic.

Out of five major suicide methods (hanging, poisoning, charcoal burning, jumping, and throwing), the SMR-S for hanging was specifically increased during the COVID-19 pandemic period compared to in the pre-pandemic period (Figure 8 and Figure 9). The annual hanging SMR-S of females in all 47 prefectures and metropolitan regions specifically increased, but significant changes in other SMR-S were not detected (Figure 8 and Figure 9). Furthermore, an increasing monthly SMR-S between the first and second stay-home orders was detected in the hanging suicide mortality of both males and females in all 47 prefectures and metropolitan regions; however, consistent changes in the other monthly SMR-S of poisoning, charcoal burning, jumping, and throwing were not observed (Figure 8 and Figure 9). Increasing and decreasing SMR-S during and after the first stay-home order, respectively, of other suicide means (poisoning, charcoal burning, jumping, and throwing) were also detected, but these fluctuations were sporadic and nonpersistent. DCP and SMR-C were positively and negatively related to the hanging SMR-S of females in both all 47 prefectures and metropolitan regions. The hanging SMR-S of females in both all 47 prefectures and metropolitan regions displayed a persistent increase from the second half of 2020 to the first quarter of 2021 (Figure 8 and Figure 9).

### 3.5. Suicide Mortality by Motive

In Japan, the most dominant suicidal motive was health-related problems (for males in order: health > economy > family > employment > romance > school; for females in order: health > family > economy > employment > romance > school) [23,24,42]. The annual SMR-S of males caused by a health- and economy-related motive in 2020 decreased, but other SMR-S caused by family-, employment-, romance-, and school-related motives did not change. The annual SMR-S of females caused by employment- and school-related motives in 2020 increased, but other SMR-S caused by family-, health-, economy-, and school-related motives did not change. In metropolitan regions, the annual SMR-S of males disaggregated by motives did not change. The annual SMR-S of females caused by employment- and romance-related motives increased, but other SMR-S disaggregated by motives did not change in metropolitan regions.

**Figure 10:**
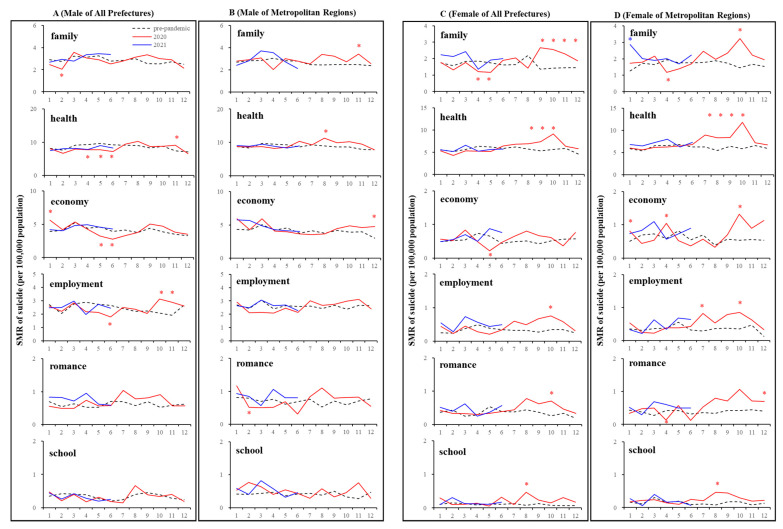
Temporal fluctuations in monthly SMR-S disaggregated by gender, suicidal reason, and regional factors during the COVID-19 pandemic period (2020–2021) compared to the average SMR-S of the same month during the pre-pandemic period (2017–2019). Dotted black, red, and blue lines indicate the average of SMR-S for (**A**) males from all 47 prefectures, (**B**) males from metropolitan regions, (**C**) females from all 47 prefectures, and (**D**) females from metropolitan regions. Ordinates indicate the SMR-S (per 100,000 people), and abscissas indicate the month. * *p* < 0.05, significant change using a linear mixed-effects model with Tukey’s multiple comparison. Red and blue asterisks indicate significant changes in SMR-S in 2020 and 2021, respectively, compared to the average SMR-S of the same month during the pre-pandemic period.

**Figure 11:**
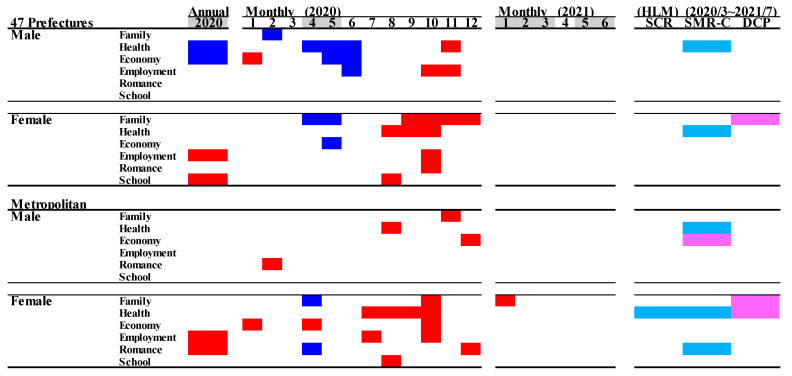
Comparison of annual and monthly SMR-S caused by suicidal motives during the pandemic period to average SMR-S of the same month during the pre-pandemic period, and impact of COVID-19 pandemic on SMR-S in Japan. Blue and red columns indicate significant decreasing and increasing suicide mortality using a linear mixed-effects model with Tukey’s multiple comparison (*p* < 0.05). Light blue and red columns indicate significant decreasing and increasing factors against SMR-S using hierarchical linear model with robust standard error (*p* < 0.05). SMR-S: standardised suicide mortality per 100,000 people. SCR: standardised infection with COVID-19 per 100,000 people. SMR-C: standardised mortality caused by COVID-19 per 100,000 people. DCP: during COVID-19 pandemic.

In all 47 prefectures, the monthly SMR-S of males caused by health-, economy-, and employment-related motives decreased during the first stay-home order. The SMR-S of females caused by family- and economy-related motives also decreased, whereas that caused by health-related motives did not during the first stay-home order (Figure 10 and Figure 11). Contrary to during the first stay-home order, between the first and second stay-home orders, female SMR-S caused by health- and family-related motives increased. Male SMR-S caused by health- and employment-related motives transiently increased, but that caused by family- and economy-related motives did not change between the first and second stay-home orders (Figure 10 and Figure 11).

In metropolitan regions, significant decreases in the monthly SMR-S of males caused by family-, health-, economy-, employment-, romance-, and school-related motives were not observed during the first stay-home order (Figure 10 and Figure 11). In the dominant SMR-S of females, the monthly SMR-S caused by family- and romance-related motives also decreased, whereas that caused by economy-related motives was unexpectedly increased during the first stay-home order. Between the first and second stay-home orders, the monthly SMR-S of males caused by health-related motives was increased, and that caused by family-, economy-, and employment-related motives transiently increased. Male SMR-S caused by health-, family-, and economy-related motives transiently increased (Figure 10 and Figure 11).

In all 47 prefectures, the hierarchical linear model detected a significant negative impact of SMR-C on the SMR-S of males and females caused by a health-related motive. The female SMR-S caused by a family-related motive was positively related to DCP (Figure 11). In metropolitan regions, the SMR-S of males caused by health-related motives was negatively related to SMR-C. Both SCR and SMR-C were negatively related to the SMR-S of females caused by health-related motives, and DCP was positively related to SMR-S caused by family- and health-related motives.

**Figure 12:**
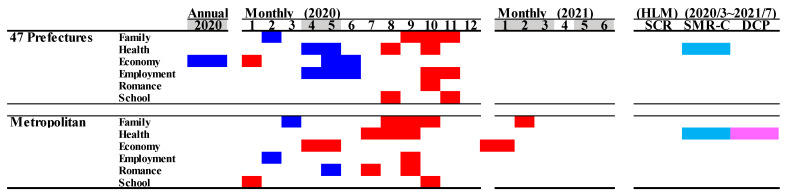
Comparison of annual and monthly SMR-S of males and females caused by suicidal motives during the pandemic period compared to average SMR-S of the same month during the pre-pandemic period, and impact of COVID-19 pandemic on SMR-S in Japan. Blue and red columns indicate significant decreasing and increasing suicide mortality using a linear mixed-effects model with Tukey’s multiple comparison (*p* < 0.05). Light blue and red columns indicate significant decreasing and increasing factors against SMR-S using hierarchical linear model with robust standard error (*p* < 0.05). SMR-S: standardised suicide mortality per 100,000 people. SCR: standardised infection with COVID-19 per 100,000 people. SMR-C: standardised mortality caused by COVID-19 per 100,000 people. DCP: during COVID-19 pandemic.

The SMR-S of females caused by economy-related motives in metropolitan regions was the sole increasing factor during the first stay-home order (Figure 10 and Figure 11). Therefore, we reanalysed the SMR-S of males and females disaggregated by five major motives. In all 47 prefectures, the SMR-S of males and females caused by economy-related motives was decreased during the first stay-home order, and increased between the first and second stay-home orders (Figure 12). In contrast, the SMR-S of males and females caused by economy-related motives was increased in May–June 2020 and January–February 2021 without decreasing in the other months (Figure 12).

## 4. Discussion

### 4.1. Overall Fluctuations of Suicide Mortality during COVID-19 Pandemic in Japan

The present study identified who died by suicide, when, why, and how during the COVID-19 pandemic period using a linear mixed-effects model and a hierarchical linear model with robust standard error. Overall fluctuations of suicide mortalities of males and females in 2020 led to a decrease during the first stay-home order (April–May 2020), and an increase between first and second stay-home orders (August–December 2020). However, the fluctuations in the suicide mortality of males and females appeared to be stabilised in the first half of 2021, including during the second (January–March 2021) and third (April–Jun 2021) stay-home orders. Contrary to national trends of suicide mortality, there was a lack of a decrease in the suicide mortality of males and females during the first stay-home order in five metropolitan regions; however, fluctuations in the suicide mortality of males and females in metropolitan regions also appeared to be stabilising in the first half of 2021. Therefore, fluctuations in suicide mortality in 2020, decreasing during the first stay-home order and increasing between the first and second stay-home orders, probably constituted a specific pattern. A recent study revealed that the risk of suicide in 21 countries could not be detected during the early COVID-19 pandemic periods. Initially, the first stay-home order was announced to metropolitan regions due to the spread COVID-19 in these regions in April 2020, but a short-term stay-home order was then announced to all 47 prefectures in May 2020 to prevent the spread of COVID-19 to rural regions induced by long holidays in May (Golden Week). Interpreted on the basis of previous natural disaster cases, the reduction in SMR-S in all 47 prefectures during the first stay-home order might be similar to the “honeymoon period” phenomenon [37,38]. However, the smaller reduction in SMR-S in the metropolitan regions during the first stay-home order cannot deny the possibility that the impact associated with COVID-19 spread or the first stay-home order on individuals in metropolitan regions was greater than that in other regions. The stabilisation of suicide mortality observed with each stay-home order may also suggest becoming accustomed to the pandemic; however, hierarchical linear model analysis detected an interaction between the negative effects of DCP (duration of the pandemic) and the positive effects of SMR-C (mortality caused by COVID-19) and SCR (infected population with COVID-19) on the suicide mortality of females, possibly neutralising each other. Therefore, suicide mortality was probably composed of various factors’ interactions.

### 4.2. When and Who Died by Suicide

This characteristic could be detected even in the issue of who died by suicide and when. Regarding the analysis of all 47 prefectures, the suicide mortality of over 40s males decreased during the first stay-home order, and did not increase between the first and second stay-home orders, whereas the suicide mortality of young (10s–20s) males did not decrease during the first stay-home order, but increased between the first and second stay-home orders. Additionally, regional characteristics of the fluctuation in males’ SMR-S in 2020 could not be detected. In the analysis of females from all 47 prefectures, the suicide mortality of young females (10s–20s) did not decrease during the first stay-home order, but increased between the first and second stay-home orders, similar to the SMR-S of young males. Contrary to males, the suicide mortality of females over 30 also decreased during the first stay-home order, but increased between the first and second stay-home orders, contrary to the suicide mortality of males over the age of 40. Regarding metropolitan regions, the SMR-S of males in metropolitan regions had a similar fluctuation pattern in all 47 prefectures; however, the SMR-S of females during the first stay-home order was minor, but increased between the first and second stay-home orders. Therefore, the combination of the increasing suicide mortality of females between the first and second stay-home orders and the less decreasing suicide mortality of females in metropolitan regions contributed to the increase in suicide mortality in 2020.

The gender-specific characteristics of SMR-S fluctuation between multiple-person household resident males and females could not be observed in all 47 prefectures, since the SMR-S of both males and females decreased during the first stay-home order and increased between the first and second stay-home orders. Contrarily, SMR-S fluctuations among one-person household resident males and females displayed a different pattern to those of multiple-person household residents, since no decrease in the suicide mortality of one-person household residents during the first stay-home order was found across all 47 prefectures. This lack of a decrease during the first stay-home order was also observed in both multiple-person and one-person household resident males and females in metropolitan regions. Therefore, the lack of a decrease in suicide mortalities among one-person household residents during the first stay-home order contributed to an increase in the annual suicide mortality of females in 2020.

### 4.3. When and Who Died by Suicide, and How

This characteristic could also be detected even in the issue of who died by suicide, when, and how. A consistent increase in hanging suicide could be detected between the first and second stay-home orders, irrespective of gender or region. Mass media frequently reported on celebrities dying by hanging suicide in July and September 2020. MHLW speculated that the increasing suicide mortality of females between the first and second stay-home orders was probably induced by these frequent reports of mass media [27,28]. In particular, these frequent reports from mass media deviated from the suicide reporting WHO guidelines “Preventing suicide: a resource for media professionals” [26]. However, the most dominant locations and tools of hanging suicide, which was the most common means of suicide in Japan [25,48], were people’s homes and every-day items, such as belts, electric flex, rafters or beams, bannisters, hooks, doorknobs, and trees [25,49]. Initially, we considered that the increase in the length of staying at home due to the stay-home order was a dominant risk factor of hanging suicide; however, the period of increasing hanging suicide mortality was not during, but following the end of the stay-home order. Although it is impossible to detect the more detailed factors behind the increasing hanging suicides in this study, it is speculated that individuals who suspended hanging suicide by the first stay-home order did die by hanging suicide due to the end of the first stay-home order, since the prolongation of the pandemic was a risk for increasing the hanging suicide mortality of females. Further analysis to identify the background factors of increasing hanging suicide using various independent variables will be published to provide important findings.

### 4.4. Who Died by Suicide, When, and Why

This characteristic could also even be detected in the issue of who died by suicide, when, and why. The most predominant suicide mortality of both males and females was caused by health-related motives [23,24,42] and was suppressed by the increasing number of COVID-19 victims (SMR-C) detected by hierarchical linear model analysis. It is easy to interpret that the lack of increasing suicide mortality of males caused by health-related motives between the first and second stay-home orders (predominant in metropolitan regions as severe infected areas with COVID-19) was sufficient to offset other increased suicides of males. Although the suicide mortality of males caused by economy-related motives in all 47 prefectures decreased during the first stay-home order, the suicide mortality of females caused by economy-related motives in metropolitan regions was unexpected to be increased in April 2020. Furthermore, in spite of lacking a significant change in males in the metropolitan region, the suicide mortality of males and females caused by economy-related motives in metropolitan regions increased during May–June 2020. The postponement of the Tokyo 2020 Olympics due to the COVID-19 pandemic was decided on 24 March 2021. The postponement decision of the Tokyo 2020 Olympics did not publish the detailed postponement date with the possible cancellation [50]. The economic effect of the Tokyo 2020 Olympics was estimated to be at least 0.2–0.3% of GDP per year [51]. This effect is particularly concentrated in the capital metropolitan area, and it can be easily estimated that the economic damages due to postponement were also large in metropolitan areas. Therefore, Japanese socioeconomic deterioration status suffered due to both the stay-home order for the suppression of COVID-19 pandemic and the postponement of the Olympics in 2020. In other words, decreasing suicide mortality in metropolitan regions during the first stay-home order was slight compared to that in all 47 prefectures, and probably generated by characteristics of urban areas and the seriousness of the COVID-19 infection situation, and by being offset through economic damages due to the postponement of the Olympics. Detailed analysis shows that the interaction between the COVID-19 pandemic and the postponement of the Olympics on economic activity in 2020 plays important roles in the clarification of suicide mortality caused by economy-related motives in capital metropolitan areas.

### 4.5. Candidate Mechanisms of Specific Fluctuations of Suicide Mortality of Younger Populations

The present study indicated several possible factors of increasing female suicide mortality in 2020. We considered that the lack of decreasing suicide mortality of young populations and one-person household resident females during the first stay-home order, and the increase after the end of the first stay-home order were characteristic fluctuation patterns of suicide mortality in 2020. A report speculated that the reason for the increased suicide mortality of young females in Japan was that the stay-home order led to an increasing unemployment rate with a decrease in temporary employment via economic recession [18]. However, the rate and number of temporary jobs between the first and second stay-home orders were deteriorated compared to the pre-pandemic period (in 2019), but this tendency was not specific to young populations [52]. Increasing domestic violence against females was a possible reason for the increasing Japanese female suicide mortality in 2020 [18], whereas instances of domestic violence events in 2020 were fewer than those in 2019 [53]. Therefore, economic recession and increasing domestic violence probably did not play important roles in the fluctuations of suicide mortality of young populations or females in 2020.

Online communication has been a standard tool in the social landscape of young populations in recent years [54]. During the pandemic, online communication became indispensable. Online communication tools played important roles in preventing isolation and maintaining schooling opportunities during the stay-home order period. However, the lengthening duration of passive social media communication in young females was related to increasing depressive symptoms [55]. Recent studies suggested that young females felt less life satisfaction and increased conflict with their parents during the COVID-19 pandemic compared to young males [56,57]. Increased family contact during the stay-home order possibly relieved the stress of young females, including academic problems, resulting in mitigating the potential negative impacts of the COVID-19 pandemic [58,59]. Therefore, young one-person-household females have probably been suffering from a vicious cycle of increasing reliance on online communication without family support. The Japan Suicide Countermeasures Promotion Center reported the possibility that the increasing suicide mortality after the end of the first stay-home order could not be explained only by the frequent suicide reports from the mass media, but also due to the combination between the large spread of the words “suicide” in SNS and the increasing suicide reports from the mass media [60]. Therefore, it is undeniable that under increasing exposure to passive online communication, the spread of the word “suicide” in SNS probably increased the risk of suicide for young one-person household resident females.

### 4.6. Limitations

There were several limitations in this study. First, BDSR published the numbers of suicide victims disaggregated by occupation; however, SBMIAC did not publish the exact occupational population as a denominator. Second, BDSR did not also publish the annual and monthly suicide mortality disaggregated by motive and age or means and age. The present study could not identify detailed background factors of increasing female suicide mortality between the first and second stay-home orders induced by these two limitations. Third, the COVID-19 pandemic is not yet over, but its impact on suicide mortality using various economic, financial, medical, and welfare indicators and comparisons of longer-term surveys of suicide mortality between Japan and other countries could identify the detailed background associated with the COVID-19 pandemic.

## 5. Conclusions

Characteristics of fluctuations in suicide mortality in Japan during the COVID-19 pandemic were outlined. Suicide mortality decreased during the first stay-home order and increased after the end of the first stay-home order. Furthermore, the direct health hazard of COVID-19 itself functioned as a suicide suppressor, but the prolongation of the COVID-19 pandemic period contributed to the increasing suicide mortality of females. Contrary to nationwide fluctuation patterns of suicide mortality, the suicide mortality of both males and females in metropolitan regions did not have a decreasing phase during the first stay-home order. Other factors, females, adolescents, one-person household residents, and metropolitan areas were possible risks of increasing suicide mortality in 2020. Additionally, the postponement of the Tokyo 2020 Olympics attenuated the decreasing suicide mortality during the first stay-home order in metropolitan regions. Taken together with previous findings associated with socioeconomic and sociopsychological deterioration, in the context of Japan, a number of reports concerned the overheated reports of mass media, financial stress, and unemployment under the governmental COVID-19 pandemic restrictions. Although suicides in Japan might have had various influences associated with the COVID-19 pandemic, these influences were consistent with complicated reasons among direct and indirect factors associated with the pandemic. Although online communication tools are important for maintaining education opportunities and preventing isolation in young populations, online communication itself possibly promotes suicide in young populations. Therefore, the enhancement of online communication tools needs to be considered as a double-edged sword for young populations.

## Figures and Tables

**Table 1:** Monthly standardised suicide mortalities (SMR-S) of males and females in all 47 prefectures and five metropolitan regions (per 100,000 people).

		All 47 Prefectures								Five Metropolitan Regions							
		Male				Female				Male				Female			
Year	Month	Mean	±	SD	(*p*)	Mean	±	SD	(*p*)	Mean	±	SD	(*p*)	Mean	±	SD	(*p*)
Pre-pandemic	1	23.2	±	4.8		9.1	±	2.2		19.2	±	3.3		8.4	±	1.4	
Average	2	20.6	±	4.3		8.6	±	2.3		21.0	±	3.3		9.6	±	1.4	
(2017–2019)	3	26.1	±	4.7		9.7	±	2.8		22.6	±	2.9		9.8	±	1.5	
	4	24.0	±	4.5		10.2	±	2.7		21.4	±	2.6		9.9	±	1.5	
	5	24.9	±	5.0		10.1	±	2.8		21.7	±	4.0		10.5	±	1.0	
	6	22.7	±	4.7		9.5	±	2.1		20.5	±	2.2		9.5	±	1.4	
	7	23.2	±	5.3		9.8	±	2.6		20.7	±	3.0		9.4	±	1.0	
	8	23.0	±	4.7		9.5	±	2.6		19.4	±	2.7		8.6	±	1.5	
	9	22.8	±	5.1		8.6	±	2.2		20.5	±	3.7		9.7	±	1.3	
	10	22.1	±	4.9		8.8	±	2.1		19.0	±	2.5		9.0	±	1.3	
	11	21.0	±	4.5		9.0	±	2.1		19.2	±	3.6		9.2	±	1.2	
	12	19.9	±	3.4		7.5	±	2.0		17.4	±	2.6		8.1	±	1.2	
2020	1	24.3	±	6.1	(0.35)	9.3	±	3.5	(0.75)	21.3	±	3.4	(0.03) *	8.4	±	1.7	(0.93)
	2	20.8	±	6.4	(0.88)	7.3	±	3.1	(0.01) **	20.1	±	3.0	(0.32)	8.6	±	2.8	(0.30)
	3	25.9	±	7.8	(0.83)	9.0	±	3.9	(0.27)	21.8	±	2.6	(0.47)	9.2	±	1.8	(0.46)
	4	21.6	±	6.1	(0.01) *	7.8	±	3.9	(0.00) **	19.0	±	3.5	(0.08)	8.9	±	1.7	(0.22)
	5	21.6	±	8.4	(0.01) **	7.9	±	3.9	(0.00) **	18.9	±	3.4	(0.11)	9.0	±	2.5	(0.08)
	6	20.7	±	5.9	(0.08)	9.7	±	3.8	(0.76)	21.1	±	3.4	(0.51)	9.7	±	1.8	(0.81)
	7	24.2	±	7.3	(0.32)	10.9	±	4.7	(0.09)	20.5	±	3.4	(0.93)	12.9	±	3.4	(0.01) **
	8	25.4	±	7.1	(0.01) *	11.7	±	4.9	(0.00) **	23.0	±	2.1	(0.00) **	11.9	±	3.2	(0.01) *
	9	25.7	±	6.1	(0.02) *	12.3	±	4.3	(0.00) **	23.0	±	3.0	(0.07)	13.4	±	1.9	(0.00) **
	10	26.7	±	6.9	(0.00) **	14.5	±	5.5	(0.00) **	24.4	±	4.4	(0.00) **	17.3	±	1.8	(0.00) **
	11	24.6	±	5.8	(0.00) **	11.4	±	4.0	(0.00) **	23.2	±	2.8	(0.01) **	11.7	±	2.8	(0.02) *
	12	21.1	±	6.3	(0.18)	10.3	±	4.6	(0.00) **	17.6	±	1.4	(0.82)	10.8	±	2.3	(0.03) *
2021	1	23.3	±	6.5	(1.00)	10.0	±	3.8	(0.37)	21.6	±	3.8	(0.03) *	11.0	±	1.6	(0.00) **
	2	23.2	±	5.5	(0.03) *	9.7	±	4.3	(0.19)	22.2	±	4.2	(0.54)	10.6	±	1.9	(0.48)
	3	26.3	±	8.3	(0.99)	12.0	±	4.6	(0.00) **	23.1	±	3.8	(0.93)	12.4	±	2.9	(0.03) *
	4	24.9	±	8.5	(0.71)	10.4	±	3.4	(0.97)	20.7	±	6.1	(0.89)	11.1	±	2.3	(0.22)
	5	24.5	±	8.2	(0.94)	10.7	±	3.5	(0.52)	19.9	±	6.4	(0.68)	9.7	±	2.7	(0.66)
	6	23.5	±	7.7	(0.80)	11.2	±	4.8	(0.06)	20.3	±	3.6	(0.99)	11.1	±	3.1	(0.25)

* *p* < 0.05, ** *p* < 0.05: significant change compared to average SMR-S of the same month during pre-pandemic period (between 2017 and 2019) using a linear mixed-effects model with Tukey’s multiple comparison. (p), *p* values of linear mixed-effects model with Tukey’s multiple comparison.

## Data Availability

All data relevant to the study are included in the article. All raw data are available to any persons via Japanese national databases from the Statistics Bureau of the Ministry of Internal Affairs and Communications (SBMIAC), the Cabinet Office (CAO), and the Ministry of Health, Labour and Welfare (MHLW).

## References

[1] World Health Organization Who Coronavirus Disease (COVID-19) Dashboard. https://covid19.who.int/.

[2] National Institute of Infectious Diseases Report Week Correspondence Table. https://www.niid.go.jp/niid/ja/calendar.html.

[3] Banerjee D., Kosagisharaf J.R., Sathyanarayana Rao T.S. (2021). ‘The dual pandemic’ of suicide and COVID-19: A biopsychosocial narrative of risks and prevention. Psychiatry Res..

[4] Gunnell D., Appleby L., Arensman E., Hawton K., John A., Kapur N., Khan M., O’Connor R.C., Pirkis J., Caine E.D.J.T.L.P. (2020). Suicide risk and prevention during the COVID-19 pandemic. Lancet Psychiatry.

[5] Kawohl W., Nordt C.J.T.L.P. (2020). COVID-19, unemployment, and suicide. Lancet Psychiatry.

[6] Reger M.A., Stanley I.H., Joiner T.E. (2020). Suicide mortality and coronavirus disease 2019—a perfect storm?. JAMA Psychiatry.

[7] Wasserman D., Iosue M., Wuestefeld A., Carli V. (2020). Adaptation of evidence-based suicide prevention strategies during and after the COVID-19 pandemic. World Psychiatry.

[8] Chang S.S., Gunnell D., Sterne J.A., Lu T.H., Cheng A.T. (2009). Was the economic crisis 1997–1998 responsible for rising suicide rates in East/Southeast Asia? A time-trend analysis for Japan, Hong Kong, South Korea, Taiwan, Singapore and Thailand. Soc. Sci. Med..

[9] Anagnostopoulos D.C., Giannakopoulos G., Christodoulou N.G. (2017). The synergy of the refugee crisis and the financial crisis in Greece: Impact on mental health. Int. J. Soc. Psychiatry.

[10] Pirkis J., John A., Shin S., DelPozo-Banos M., Arya V., Analuisa-Aguilar P., Appleby L., Arensman E., Bantjes J., Baran A. (2021). Suicide trends in the early months of the COVID-19 pandemic: An interrupted time-series analysis of preliminary data from 21 countries. Lancet Psychiatry.

[11] John A., Eyles E., Webb R.T., Okolie C., Schmidt L., Arensman E., Hawton K., O’Connor R.C., Kapur N., Moran P. (2020). The impact of the COVID-19 pandemic on self-harm and suicidal behaviour: Update of living systematic review. F1000Research.

[12] Rogers J.P., Chesney E., Oliver D., Begum N., Saini A., Wang S., McGuire P., Fusar-Poli P., Lewis G., David A.S. (2021). Suicide, self-harm and thoughts of suicide or self-harm in infectious disease epidemics: A systematic review and meta-analysis. Epidemiol. Psychiatr. Sci..

[13] Ministry of Health, Law Basic Data on Suicide in the Region. https://www.mhlw.go.jp/stf/seisakunitsuite/bunya/0000140901.html.

[14] Fushimi M. (2021). The importance of studying the increase in suicides and gender differences during the COVID-19 pandemic. QJM Int. J. Med..

[15] Sakamoto H., Ishikane M., Ghaznavi C., Ueda P. (2021). Assessment of Suicide in Japan during the COVID-19 Pandemic vs Previous Years. JAMA Netw. Open.

[16] Nomura S., Kawashima T., Harada N., Yoneoka D., Tanoue Y., Eguchi A., Gilmour S., Kawamura Y., Hashizume M. (2021). Trends in suicide in Japan by gender during the COVID-19 pandemic, through December 2020. Psychiatry Res..

[17] Seposo X.T. (2021). COVID-19 threatens decade-long suicide initiatives in Japan. Asian J. Psychiatr..

[18] Eguchi A., Nomura S., Gilmour S., Harada N., Sakamoto H., Ueda P., Yoneoka D., Tanoue Y., Kawashima T., Hayashi T.I. (2021). Suicide by gender and 10-year age groups during the COVID-19 pandemic vs previous five years in Japan: An analysis of national vital statistics. Psychiatry Res..

[19] Tanaka T., Okamoto S. (2021). Increase in suicide following an initial decline during the COVID-19 pandemic in Japan. Nat. Hum. Behav..

[20] Kino S., Jang S.N., Gero K., Kato S., Kawachi I. (2019). Age, period, cohort trends of suicide in Japan and Korea (1986–2015): A tale of two countries. Soc. Sci. Med..

[21] Kato R., Okada M. (2019). Can Financial Support Reduce Suicide Mortality Rates?. Int. J. Environ. Res. Public Health.

[22] Okada M., Hasegawa T., Kato R., Shiroyama T. (2020). Analysing regional unemployment rates, GDP per capita and financial support for regional suicide prevention programme on suicide mortality in Japan using governmental statistical data. BMJ Open.

[23] Nakamoto M., Nakagawa T., Murata M., Okada M. (2021). Impacts of Dual-Income Household Rate on Suicide Mortalities in Japan. Int. J. Environ. Res. Public Health.

[24] Shiroyama T., Fukuyama K., Okada M. (2021). Effects of Financial Expenditure of Prefectures/Municipalities on Regional Suicide Mortality in Japan. Int. J. Environ. Res. Public Health.

[25] Hasegawa T., Matsumoto R., Yamamoto Y., Okada M. (2021). Analysing effects of financial support for regional suicide prevention programmes on methods of suicide completion in Japan between 2009 and 2018 using governmental statistical data. BMJ Open.

[26] Ministry of Health, Labor and Welfare Preventing Suicide: A Resource for Media Professionals-Update 2017. https://www.who.int/mental_health/suicide-prevention/resource_booklet_2017/en/.

[27] Ministry of Health, Labor and Welfare Survey Study on the Actual Conditions of Young Carers. https://elaws.e-gov.go.jp/search/elawsSearch/elaws_search/lsg0500/detail?lawId=501AC1000000032_20190912_000000000000000&openerCode=1.

[28] Ministry of Health, Labor and Welfare Calling Attention When Reporting on Celebrity Suicide. https://www.mhlw.go.jp/stf/seisakunitsuite/bunya/hukushi_kaigo/seikatsuhogo/jisatsu/who_tebiki.html.

[29] Wasserman I.M. (1992). The impact of epidemic, war, prohibition and media on suicide: United States, 1910–1920. Suicide Life Threat. Behav..

[30] Liang S.T., Liang L.T., Rosen J.M. (2021). COVID-19: A comparison to the 1918 influenza and how we can defeat it. Postgrad. Med. J..

[31] Chan S.M., Chiu F.K., Lam C.W., Leung P.Y., Conwell Y. (2006). Elderly suicide and the 2003 SARS epidemic in Hong Kong. Int. J. Geriatr. Psychiatry.

[32] Cheung Y.T., Chau P.H., Yip P.S. (2008). A revisit on older adults suicides and Severe Acute Respiratory Syndrome (SARS) epidemic in Hong Kong. Int. J. Geriatr. Psychiatry.

[33] Yip P.S., Cheung Y.T., Chau P.H., Law Y.W. (2010). The impact of epidemic outbreak: The case of severe acute respiratory syndrome (SARS) and suicide among older adults in Hong Kong. Crisis.

[34] Zortea T.C., Brenna C.T.A., Joyce M., McClelland H., Tippett M., Tran M.M., Arensman E., Corcoran P., Hatcher S., Heisel M.J. (2020). The Impact of Infectious Disease-Related Public Health Emergencies on Suicide, Suicidal Behavior, and Suicidal Thoughts. Crisis.

[35] Fire and Disaster Management Agency 2014 Firefighting White Paper. https://www.fdma.go.jp/publication/#whitepaper.

[36] Matsubayashi T., Sawada Y., Ueda M. (2013). Natural disasters and suicide: Evidence from Japan. Soc. Sci. Med..

[37] Madianos M.G., Evi K. (2010). Trauma and natural disaster: The case of earthquakes in Greece. J. Loss Trauma.

[38] Gordon K.H., Bresin K., Dombeck J., Routledge C., Wonderlich J.A.J.C. (2011). The impact of the 2009 Red River Flood on interpersonal risk factors for suicide. Crisis.

[39] Farooq S., Tunmore J., Ali W., Ayub M. (2021). Suicide, self-harm and suicidal ideation during COVID-19: A systematic review. Psychiatry Res..

[40] John A., Pirkis J., Gunnell D., Appleby L., Morrissey J. (2020). Trends in suicide during the COVID-19 pandemic. BMJ.

[41] Statistics Bureau of the Ministry of Internal Affairs and Communications Surveys of Population, Population Change and the Number of Households Based on the Basic Resident Registration. https://www.e-stat.go.jp/stat-search/files?page=1&toukei=00200241&tstat=000001039591.

[42] Nakano T., Hasegawa T., Okada M. (2021). Analysing the Impacts of Financial Support for Regional Suicide Prevention Programmes on Suicide Mortality Caused by Major Suicide Motives in Japan Using Statistical Government Data. Int. J. Environ. Res. Public Health.

[43] Shiratori Y., Tachikawa H., Nemoto K., Endo G., Aiba M., Matsui Y., Asada T. (2014). Network analysis for motives in suicide cases: A cross-sectional study. Psychiatry Clin. Neurosci..

[44] Idogawa M., Tange S., Nakase H., Tokino T. (2020). Interactive Web-based Graphs of Coronavirus Disease 2019 Cases and Deaths per Population by Country. Clin. Infect. Dis..

[45] Fukuyama K., Kato R., Murata M., Shiroyama T., Okada M. (2019). Clozapine Normalizes a Glutamatergic Transmission Abnormality Induced by an Impaired NMDA Receptor in the Thalamocortical Pathway via the Activation of a Group III Metabotropic Glutamate Receptor. Biomolecules.

[46] Nakano T., Hasegawa T., Suzuki D., Motomura E., Okada M. (2019). Amantadine Combines Astroglial System Xc(-) Activation with Glutamate/NMDA Receptor Inhibition. Biomolecules.

[47] Okada M., Fukuyama K., Kawano Y., Shiroyama T., Ueda Y. (2019). Memantine protects thalamocortical hyper-glutamatergic transmission induced by NMDA receptor antagonism via activation of system xc. Pharmacol. Res. Perspect..

[48] Ministry of Health, Labor and Welfare 2020 White Paper on Suicide Prevention. https://www.mhlw.go.jp/stf/seisakunitsuite/bunya/hukushi_kaigo/seikatsuhogo/jisatsu/jisatsuhakusyo2020.html.

[49] Gunnell D., Bennewith O., Hawton K., Simkin S., Kapur N. (2005). The epidemiology and prevention of suicide by hanging: A systematic review. Int. J. Epidemiol..

[50] IOC Joint Statement from the International Olympic Committee and the Tokyo 2020 Organising Committee. https://olympics.com/ioc/news/joint-statement-from-the-international-olympic-committee-and-the-tokyo-2020-organising-committee.

[51] Nagata M., Ojima M., Kurachi T., Miura H., Kawamoto T. (2015). Economic effects of the 2020 Tokyo Olympics (Japanese). Bank Jpn. Rep. Res. Pap..

[52] Statistics Bureau of the Ministry of Internal Affairs and Communications Labor Force Survey. https://www.stat.go.jp/data/roudou/pref/index.html.

[53] Agency N.P. Criminal Statistics: Stoker and Domestic Violence. https://www.npa.go.jp/publications/statistics/safetylife/dv.html.

[54] Odgers C.L., Schueller S.M., Ito M. (2020). Screen time, social media use, and adolescent development. Annu. Rev. Dev. Psychol..

[55] Ellis W.E., Dumas T.M., Forbes L.M. (2020). Physically isolated but socially connected: Psychological adjustment and stress among adolescents during the initial COVID-19 crisis. Can. J. Behav. Sci. /Rev. Can. Des. Sci. Du Comport..

[56] Kapetanovic S., Gurdal S., Ander B., Sorbring E. (2021). Reported changes in adolescent psychosocial functioning during the COVID-19 outbreak. Adolescents.

[57] Magson N.R., Freeman J.Y.A., Rapee R.M., Richardson C.E., Oar E.L., Fardouly J. (2021). Risk and Protective Factors for Prospective Changes in Adolescent Mental Health during the COVID-19 Pandemic. J. Youth Adolesc..

[58] Fegert J.M., Vitiello B., Plener P.L., Clemens V. (2020). Challenges and burden of the Coronavirus 2019 (COVID-19) pandemic for child and adolescent mental health: A narrative review to highlight clinical and research needs in the acute phase and the long return to normality. Child. Adolesc. Psychiatry Ment. Health.

[59] Hoekstra P.J. (2020). Suicidality in children and adolescents: Lessons to be learned from the COVID-19 crisis. Eur. Child. Adolesc. Psychiatry.

[60] Japan Suicide Countermeasures Promotion Center Enlightenment/Recommendation. https://jscp.or.jp/action/jisatsu_benkyokai_report0810.html.

